# Reviewer acknowledgement

**DOI:** 10.1186/s13064-016-0061-2

**Published:** 2016-03-08

**Authors:** Chris Q. Doe, William Harris, Kang Shen, Rachel Wong

**Affiliations:** University of Oregon, Eugene, USA; University of Cambridge, Cambridge, England UK; Stanford University, Stanford, California USA; University of Washington, Seattle, Washington USA

## Abstract

The editors of *Neural Development* would like to thank all the reviewers who have contributed to the journal in Volume 10 (2015).

**Richard Adams**

United Kingdom

**Christine Aurich**

Austria

**Clare Baker**

United Kingdom

**Anthony Barnes**

United States of America

**Marie-Laure Baudet**

Italy

**Eric Bellefroid**

Belgium

**Olivia Bermingham-Mcdonogh**

United States of America

**Florence Besse**

France

**Holger Bielen**

United Kingdom

**Sandra Blaess**

Germany

**Paola Bovolenta**

Spain

**Andrea Brand**

United Kingdom

**Susan Brockerhoff**

United States of America

**Jean-Francois Brunet**

France

**Florencia Cavodeassi**

Spain

**Chieh Chang**

United States of America

**Zhe Chen**

United States of America

**Ajay Chitnis**

United States of America

**Hollis Cline**

United States of America

**Helen Cooper**

Australia

**David Copenhagen**

United States of America

**Robin Davis**

United States of America

**Christopher Deppmann**

United States of America

**Chris Doe**

United States of America

**Richard Dorsky**

United States of America

**Kazuo Emoto**

Japan

**Edward Giniger**

United States of America

**Tom Glaser**

United States of America

**Phillip Gordon-Weeks**

United Kingdom

**Yukiko Gotoh**

Japan

**Elizabeth Ann Grove**

United States of America

**Francois Guillemot**

United Kingdom

**Kurt Haas**

Canada

**Mark Hankins**

United Kingdom

**Yi-Ping Hsueh**

Taiwan, Republic of China

**Lilia Iakoucheva**

United States of America

**Hiroyuki Kamiguchi**

Japan

**Karl Kandler**

United States of America

**Artur Kania**

Canada

**Tim Kennedy**

Canada

**Daniel Kerschensteiner**

United States of America

**Lawrence Kromer**

United States of America

**Raj Ladher**

India

**Cheng-Yu Lee**

United States of America

**James Li**

United States of America

**Rick Livesey**

United Kingdom

**Hernan Lopez-Schier**

Germany

**Xiaowei Lu**

United States of America

**Erik Lundquist**

United States of America

**Le Ma**

United States of America

**Ryan Macdonald**

United Kingdom

**Jeremy Nathans**

United States of America

**Kevin Park**

United States of America

**Muriel Perron**

France

**Stephen Price**

United Kingdom

**Jonathan Raper**

United States of America

**Anne Ridley**

United Kingdom

**Jean-Yves Roignant**

Germany

**David Rowitch**

United States of America

**Dietmar Schmucker**

Belgium

**Carol Schuurmans**

Canada

**Jerry Silver**

United States of America

**Patrice Smith**

United Kingdom

**David Solecki**

United States of America

**Florentina Soto**

United States of America

**Kristin Tessmar-Raible**

Austria

**David Van Vactor**

United States of America

**Wei Wei**

United States of America

**Donna Whitlon**

United States of America

**Hynek Wichterle**

United States of America

**Miles Wilkinson**

United States of America

**Kevin Wright**

United States of America

**Fengwei Yu**

Singapore

**Chun-Li Zhang**

United States of America

**Kai Zinn**

United States of America

